# Is 8:30 a.m. Still Too Early to Start School? A 10:00 a.m. School Start Time Improves Health and Performance of Students Aged 13–16

**DOI:** 10.3389/fnhum.2017.00588

**Published:** 2017-12-08

**Authors:** Paul Kelley, Steven W. Lockley, Jonathan Kelley, Mariah D. R. Evans

**Affiliations:** ^1^Sleep, Circadian and Memory Neuroscience, The Open University, Milton Keynes, United Kingdom; ^2^Division of Sleep and Circadian Disorders, Brigham and Women's Hospital, Boston, MA, United States; ^3^Division of Sleep Medicine, Harvard Medical School, Harvard University, Boston, MA, United States; ^4^International Survey Center, Sociology, University of Nevada, Reno, NV, United States; ^5^Sociology and Applied Statistics Program, University of Nevada, Reno, NV, United States

**Keywords:** school start times, sleep, circadian, illness, academic performance, adolescence, circadian social science

## Abstract

While many studies have shown the benefits of later school starts, including better student attendance, higher test scores, and improved sleep duration, few have used starting times later than 9:00 a.m. Here we report on the implementation and impact of a 10 a.m. school start time for 13 to 16-year-old students. A 4-year observational study using a before-after-before (A-B-A) design was carried out in an English state-funded high school. School start times were changed from 8:50 a.m. in study year 0, to 10 a.m. in years 1–2, and then back to 8:50 a.m. in year 3. Measures of student health (absence due to illness) and academic performance (national examination results) were used for all students. Implementing a 10 a.m. start saw a decrease in student illness after 2 years of over 50% (*p* < 0.0005 and effect size: Cohen's *d* = 1.07), and reverting to an 8:50 a.m. start reversed this improvement, leading to an increase of 30% in student illness (*p* < 0.0005 and Cohen's *d* = 0.47). The 10:00 a.m. start was associated with a 12% increase in the value-added number of students making good academic progress (in standard national examinations) that was significant (<0.0005) and equivalent to 20% of the national benchmark. These results show that changing to a 10:00 a.m. high school start time can greatly reduce illness and improve academic performance. Implementing school start times later than 8:30 a.m., which may address the circadian delay in adolescents' sleep rhythms more effectively for evening chronotypes, appears to have few costs and substantial benefits.

## Introduction

Despite the well-established natural shifts to later wake and sleep times that occur in adolescence, most schools retain early start times. Currently school starting times are not adjusted for the shift to later wake and sleep times that occur naturally in adolescence. This mismatch between adolescent biology and the conventional practice of starting school early leads to a systematic reduction in the amount of time available for sleep to teenagers and consequently chronic sleep deficiency. The Centers for Disease Control and Prevention and the American Academy of Pediatrics (2014) have stated that early school starts are associated with increased health risks of obesity, depression, and drug use as well as poorer academic performance (Owens et al., 2014; Wheaton et al., 2015). Their recommendation that middle and high schools should open no earlier than 8:30 a.m. is now supported by the American Medical Association (2016). Almost all studies to date, while scheduling school starting times later than before, have retained a starting time at or earlier than 9:00 a.m. (Kirkby et al., 2011). One study in New Zealand did report improved sleep in 17 to 18-year-old students when the starting time was moved from 9:00 to 10:30 a.m., as compared to younger controls who remained at 9:00 a.m. (Borlase et al., 2013). A recent study of optimal times for cognitive performance for students aged 18–19 concluded that much later times were optimal, specifically after 11 a.m. or 12 noon (Evans et al., 2017).

Evidence for increased health risks associated with early school starting times is both substantial and demonstrated through a variety of research methodologies (Hansen et al., 2005; Millman, 2005; Basch et al., 2014; de Souza and Hidalgo, 2014). The underlying biological drivers are also well established. Adolescents need anywhere from 8 to 10 h of sleep per night for full health and academic performance depending on age and inter-individual differences, yet most get far less (Iglowstein et al., 2003; Foster et al., 2013; Hirshkowitz et al., 2015). Biological changes in the timing of the 24-h circadian clock during adolescence delay the onset of wake and sleep times, and this shift does not reverse until early adulthood (Roenneberg et al., 2004). Additionally, homeostatic regulation of pressure to sleep builds more slowly, taking a longer time to reach the critical threshold required to initiate sleep (Carskadon, 2011). Adolescent sleep restriction is clearly linked to early school starts as on non-school days adolescents have wake times two or more hours later (Roenneberg et al., 2007), a finding seemingly not substantially affected by cultural factors (Gradisar et al., 2011; Foster et al., 2013).

Later school starting times provide benefits to adolescent sleep, health and learning (Curcio et al., 2006; Carskadon, 2011; Lufi et al., 2011). There is substantial evidence that later starting times benefit academic outcomes even in the early stages of puberty and this positive impact continues into late adolescence (Carrell et al., 2011; Edwards, 2012; Meltzer et al., 2014). Later starting times are associated with improved sleep that continues into the years following implementation (Borlase et al., 2013; Wahlstrom et al., 2014). Later starting times also reduce the rate of student driving accidents (Danner and Phillips, 2008), and lower reports of depression (Kirkby et al., 2011).

The principle that school start times for adolescents should be later than currently the norm in American schools (about 8:00 a.m.) is now widely accepted. Research is now needed into synchronizing school starting times more closely with adolescent biology, taking into account the increasing impact of circadian rhythm changes in adolescence (Shekleton et al., 2013; Kelley et al., 2015). While the evidence for starting middle and high schools at 8:30 a.m. or later is positive, this recommendation is based on evidence limited to studies where school times are shifted to no later than 9:00 a.m., or often earlier, leaving unanswered the question of how late school starting times should be. To explore the impact of a much later school start time, we examined the impact of a 10 a.m. starting time (representing a 1:10 h delay from an 8:50 a.m. starting time), on rates of illness of 13 to 16-year-old students and academic performance of 14 to 16-year-old students.

## Participants and methods

### Determining the start time

In light of the then-existing evidence on start times, the lead investigator of the present study (PK) designed and directed a field experimental to implement and assess the efficacy of a much later school start time. The design is summarized in Table 1, columns 1, 2, and 4 (see section Results). The school was a mixed sex, state-funded school's for 13 to 18-year-old students. At the start of the study, the school student performance was considerably below the national mean. The school was in an urban area of 0.7 million, in a region of England where achievement was lower than national average.

**Table 1:**
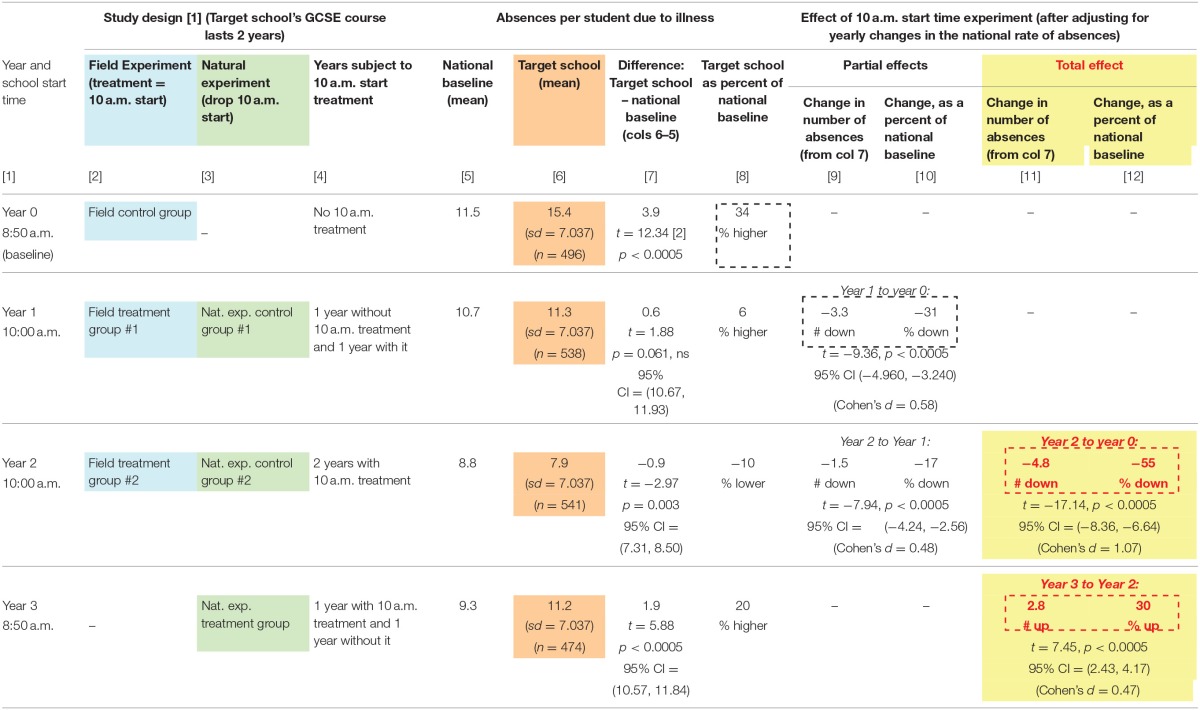
Effect of experimental treatment (10 a.m. school start time) on absences due to illness.

*Key results (columns 11 and 12) highlighted in red boldface*.

*[1] The field experiment was designed and directed by one of the authors (PK). The natural experiment was the result of a change in control of the target school to local government that reinstated the early start in that was used in all local schools*.

*[2] Raw data are not publicly available and standard deviations are not published. We use here an estimate of the standard deviation based on the grouped percentage distribution of absences. Assuming uniform distributions within intervals gives a conservative (wider) IQR. Then assuming absences are approximately normally distributed, we take IQR/1.35 = estimated standard deviation. Here that is 7.037*.

The school had no additional funding, selective entry or other educational interventions during the duration of the study. The school operated within The Innovation Trust, a charitable trust to improve schools in partnership with leading scientists (see section Acknowledgments). Working with this group of experts, a later start time was considered with a focus on determining a starting time for adolescents aged 13–18. Initial testing of student academic performance at 10:00 and 14:00, voluntary chronotype surveys and body fat levels of students led to the conclusion there should be a core time period for all students between 11:00 and 15:00, with additional learning time before (for morning/average chronotypes) and after (for evening chronotypes) (Sussman, 2009). This appeared too radical a change and, combined with the then current National Health Service Trust judgement that schools could not legally hold any mental health data on students 16 or under including sleep and body fat data, led to limiting the age range of 13–16 and using only existing national measures of health and academic performance. Therefore analysis of the relevant sleep and circadian neuroscience research indicating 10:00–10:30 a.m. starting times would be appropriate at age 16 (Kelley et al., 2015). This process led to the final choice of 10:00 a.m. as the school start time, a 1 h 10 min delay compared to the traditional 8:50 a.m. start time then in use.

The 2-year process for changing start times was ethically approved by both The Innovation Trust, a charitable educational research trust linked to the school, and the school's Governing Body on the basis that students would not be subject to additional surveys, tests or measures other than a later starting time. A legally-required consultation with parents, students, teachers and others was conducted. This raised a number of concerns including transport to school. Unlike American schools, urban schools in England generally do not provide transport for all students, and the existing transport infrastructure made this a relatively minor issue. The school already supported parental work hours and transport by opening 1 h early and 1 h after school (this included sports and club opportunities). Therefore, with minor changes, all the times in the school were simply moved an hour and 10 min later. Although parents supported the changes, there was resistance from the external local educational administrators, a phenomenon noted in other studies (Wahlstrom et al., 2014). The concerns raised during the consultation, while often valid, were addressed as possible, but considered secondary to the raison d'etre for schools—to help healthy children realize their full academic potential.

After approval of the change, a 6-week trial run with a 10:00 a.m. start was conducted in 2010 after all annual examinations were completed and students taking examinations had left, but before the start of the new academic year. The trial moved all related scheduled events to match the 10:00 a.m. start including transport to and from school, family arrangements, and school clubs and activities. There were also some additional and unexpected benefits reported: The travel times avoided rush hour and were considered safer; and some staff could take their children to their primary school in the morning.

After 2 years with the 10 a.m. start time, a natural experiment was created by a change in local education administrators that reinstated the early start as used in all local schools. Consequently, the school starting time for 13 to 16-year olds was changed back to 8:50 a.m. due to these policy changes, not part of the study design; however we took advantage of this natural experiment.

### Intervention and data collection

This observational study used a before-after-before (A-B-A) design. School start times changed as follows over 4 years: Year 0 had an 8:50 a.m. start; Years 1 and 2 had a 10:00 a.m. start; and Year 3 had an 8:50 a.m. start. Over these four academic years (September 2010–August 2014) national data and school data on illness and performance of 2,049 students aged 13–16 years were collected.

English education data are collected nationally for each school and these data for all schools are published by the UK Office of National Statistics (2016). Two variables were used to assess the impact of the time change: Student absence due to illness, and overall student academic performance. English schools are required to distinguish absence (not in school for any reason) from absence due to illness (which accounts for ~60% of all absences, varying by year). Absence due to illness is recorded for each student for every morning and afternoon session, and so the measure of illness utilized here was the number of school sessions lost through illness per student per year. This measure gives a more precise measure of illness than raw absences rates (Department for Education, 2016).

At that time, the government's key measure of student academic performance was the General Certificate of Secondary Education (GCSE) examinations taken at the end of compulsory schooling at age 16. Good academic progress at 16 was defined as achieving five or more GCSE grades of C or above in English, Mathematics and at least three other subjects. The measure of school performance was the percentage of students achieving good academic progress.

In addition, a national system of value-added analysis of individual students and school potential achievement in GCSE grades and good academic progress was produced by the Fisher Family Trust^1^ for all students and schools in England. As the prior ability of student cohorts can vary year-to-year, so can the effectiveness of schools, and thus a second analysis of performance based on these value-added predictions was undertaken (Visscher and Coe, 2003; Koedel et al., 2015). The predicted results were based on the students' previous achievement in national tests. Schools exceeding predicted percentage good academic progress were deemed to have added value, given as a percentage good academic progress above the predicted outcomes (with negative results for schools below predictions). For example, if the students achieved an average of 4% higher good academic progress, it would have a value-added score of +4%. These two national measures of actual and predicted school performance were used in our analyses.

### Analyses

National data were used to analyse illness and performance. National data were used where available to estimate standard deviations. In addition, a data set of 2,880 similar schools was constructed where “similar” was defined as state-funded school with 13 to 16-year-old students in cohorts >100, and reporting examination data for the period 2010–2014. The data sets used are school-level data published by the UK's Office of National Statistics and other sources as indicated (UK Office of National Statistics, 2016). The achievement data have limitations due to policy and publication practices. Policy limitations include a decision to remove schools becoming academies (similar to Charter schools) from national data, changes in examinations, and in the data reported during the 4 years.

Analysis of illness and performance data during this period led to reporting both raw scores and scores adjusted to account for national variation. For national trends in illness, performance, *T*-tests were used to assess significance, and Cohen's d and h for effect size, taking into account Hattie's critique of educational measures to determine impact of interventions (Hattie, 2008). Hattie's critique of educational research based on synthesizing over 800 meta-analyses on raising achievement raised questions about educational data analyses and significance. Specifically, he argued that more sophisticated statistical techniques, large numbers of subjects usually possible within education research, and other factors meant effect sizes should be >0.5 in individual studies. Hattie lists 138 effect sizes found in meta-analyses of the significant educational interventions of the last 20 years, showing the highest 20 interventions had effect sizes ranging from 0.61 to 1.44. Hattie's concerns about educational research and effect sizes are shared by others (Snow, 2015; Churches et al., 2017). For illustrative purposes, the cost/ benefits of educational reforms in England and New York to increase educational performance was also analyzed.

## Results

**Figure 1:**
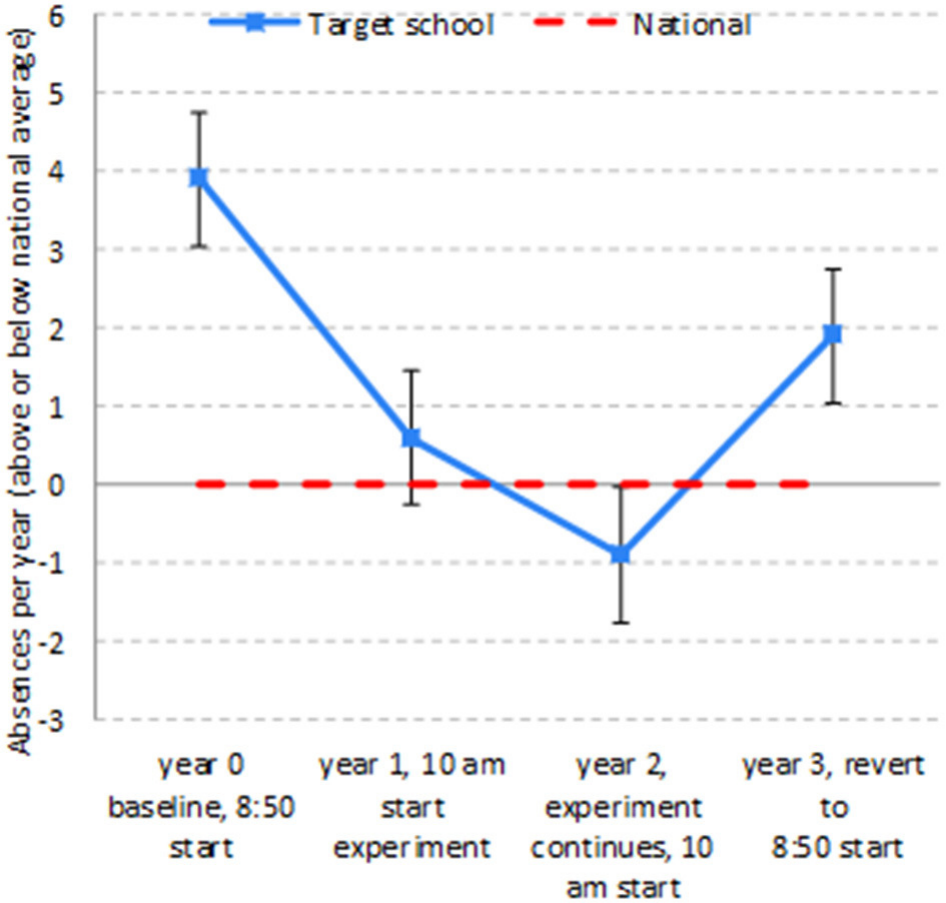
Absences due to illness, differences from national average.

Rates of absence due to illness of students aged 13–16 were lower with the 10 a.m. start time (see Table 1 and Figure 1).

In Year 0, for the baseline cohort of students aged 13–16 which had experienced 8:50 start times for both years of their course in the target school, the mean number of absences due to illness was 15.4, compared to 11.5 nationally (Year 0 “Field Control Group” in Table 1, compare column 6 for the target school to column 5 for the national benchmark). That is a difference of 3.9 absences which is statistically significant at *p* < 0.0005 (column 7). Moreover, it is large: The rate in the target school was 34% higher than in the national benchmark (column 8).

The situation changed sharply for Year 1 (“Field Treatment group #1” in Table 1), the first year with the experimental 10 a.m. start treatment. The mean number of absences in the target school dropped from 15.4 to 11.3, a drop of 4.1 absences (column 6, compare Year 0 and Year 1). The gap between the target school absence rate and the national benchmark shrank by 3.3 absences to a mere 0.6 absences (Year 1, column 7). This shrinkage amount to a 31% decline in the gap between the target school and the national benchmark (Year 1, column 10), a decline that is statistically significant at *p* < 0.0005. Cohen's d for the change is 0.58, making the effect size in the medium-to-large range. This reduced absence rate left the target school a mere 0.6 absences above the national benchmark, a difference so small that it is not statistically significant (*p* > 0.05, column 7).

The crux of the field experiment is Year 2, when the 10 a.m. start was operating for its second full year (the target school's GCSE courses last 2 years with examinations throughout). Absences dropped further in the target school, from a mean of 11.3 to a mean of 7.9 (column 6), a drop of 3.4 absences. This brought the target school's absence rate significantly below the national benchmark (column 7). This shift from slightly above the national benchmark in Year 1 to significantly below the national benchmark in Year 2 has a Cohen's d of 0.48, a medium effect size.

The most crucial comparison is between Year 2 when the 10 a.m. start was operating for its second full year and Year 0 in which students had been on the 8:50 start for at least 2 years. The mean number of absences fell by fully 7.5 (Column 6, Year 2 vs. Year 0). The gap between the target school rate and the national benchmark shrank by 55%, amounting to a shrinkage of 4.8 absences. This change is statistically significance at *p* < 0.0005. The Cohen's d for the change is 1.07, a large effect size.

Thus, the field experiment demonstrates in multiple ways that the experimental treatment is associated with a decline in absences that is large and statistically significant not only relative to the control group but also relative to the national benchmark. The pattern is evident in Figure 1.

Next comes the natural experiment when the start time in the target school returned to 8:50 in Year 3 due to a change in policy which shifted control of the target school to local education authorities who imposed a uniform early start time on all comparable schools. The mean number of absences rebounded to 11.2, well above the level in the previous year (column 6). This was 20% above the national benchmark, a clear contrast with the previous year's 10% below the benchmark (column 8). Adjusting for yearly changes in the national rate of absences, the gap between the target school and the nation inflated to 2.8 absences a year, a 30% increase (columns 11 and 12). This change is statistically significant at *p* < 0.0005 and has a Cohen's d of 0.47, a medium effect size.

All in all, for absences the field experiment demonstrates that the change to a 10 a.m. start reduced absences due to illness by over 50 percent compared to national rates, a large and statistically significant decline. The natural experiment demonstrates that reverting to the 8:50 start induced, already in its first year, a medium sized, statistically significant increase in absences. These data are consistent with a dose-dependent response to a 10:00 start.

In the target school's Year 0 (“Field Control Group” in Table 2) the baseline cohort of students which had experienced 8:50 start times for both years of their course, the percentage who successfully completed the GCSE examinations was 34, compared to 56.2 nationally (columns 5 and 6). That is a vast difference of 22 percentage points, statistically significant at *p* < 0.0005 (column 7). This difference amounts to 40% of the national baseline (column 8), marking the target school as having a very low success rate. In value added terms, the actual percentage of successful students was 5 percentage points lower than the FFT estimate for the school based on the cohort's past performance (column 9), showing a negative value-added value. All in all, in Year 0 in the target school, the performance picture was grim.

**Table 2:**
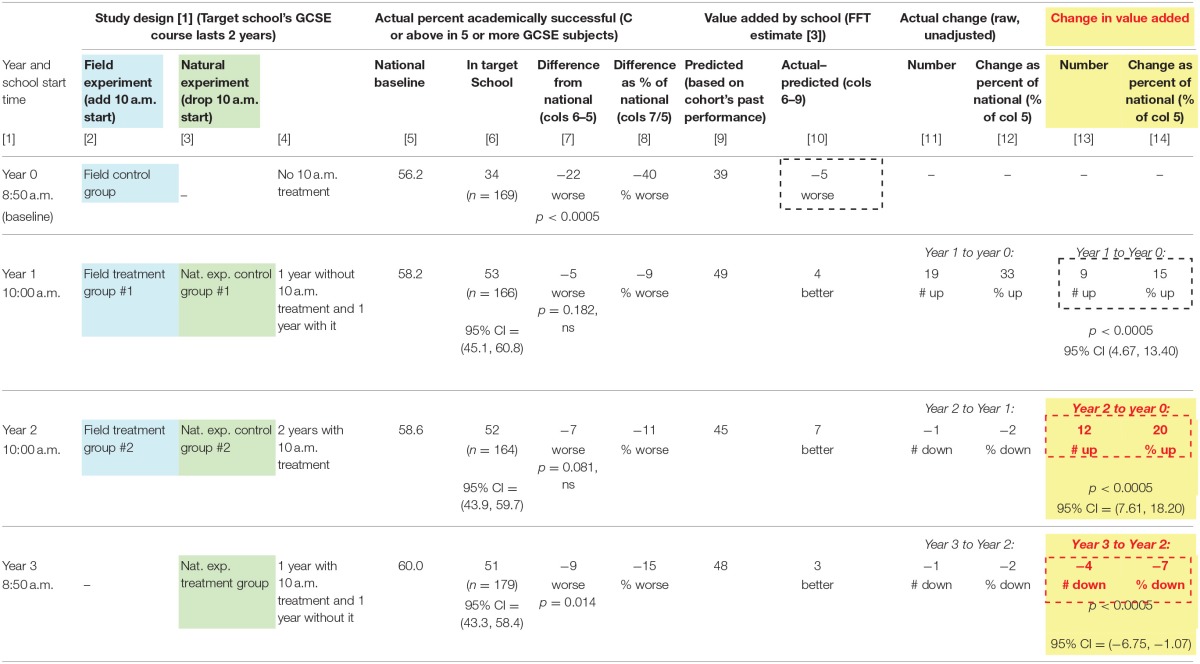
Effect of experimental treatment (10 a.m. school start time) on academic success.

*Success is defined, following government convention, as achieving a grade of C or above in five or more subjects in the national General Certificate of Secondary Education (GCSE) examinations. Key results (columns 13 and 14) highlighted in red boldface*.

*[1] The field experiment was designed and directed by one of the authors (PK). The natural experiment was the result of a change in control of the target school to local government that reinstated the early start in that was used in all local schools*.

*[2] Raw data are not publicly available and standard deviations are not published. We use here an estimate of the standard deviation based on the grouped percentage distribution of absences. Assuming uniform distributions within intervals gives a conservative (wider) IQR. Then assuming absences are approximately normally distributed, we take IQR/1.35 = estimated standard deviation. Here that is 7.037*.

*[3] Fisher Family Trust Value-added estimates, widely used in British education (https://fft.org.uk)*.

The situation changed sharply for Year 1, the first year with the experimental 10 a.m. start treatment. The actual percent of academically successful students shot up by 19 percentage points to 53 (column 6, compare Year 0 and Year 1). The gap between the target school and in national benchmark in Year 1 is so small that it is not statistically significant (Year 1, column 7). Between Year 0 and Year 1, the gap between the target school success rate and the national benchmark shrank from 22 to 5 (Year 1, column 7), a shrinkage of 17 percentage points that is statistically significant at *p* < 0.0005. The value added by the school has also risen: The actual value added by the school is 4 percentage points more than the FFT prediction, up 9 percentage points from Year 0. This amounts to 15% of the national baseline and is statistically significant at *p* < 0.0005.

The heart of the field experiment is the comparison of Year 2 vs. Year 0. Here again there is extensive evidence for the positive impact of the 10 a.m. start on student success. Student success in Year 2 is 52% compared to 34% for Year 0 (column 6), a gain which is significant at *p* = 0.001 and of medium size, with a Cohen's H of 0.37. Between Year 0 and Year 2, the gap between the target school success rate and the national benchmark shrank from 22 to 7 (Year 2, column 7). The actual student success in Year 2 exceeds the FFT prediction by 7 percentage points (column 10). Indeed, there is a 12-percentage point gain in the value added by the school, a gain that is statistically significant at *p* < 0.0005 (column 13) and amounts to 20% of the national benchmark.

Thus, the field experiment demonstrates in multiple ways that the experimental treatment is associated with a statistically significant and substantial gain in performance not only relative to the control group but also relative to the national benchmark.

Then, in the natural experiment in Year 3, the school starting time reverted from 10 a.m. to 8:50 a.m. The student success rate fell slightly to 51 (Year 3, column 6) in comparison to a national benchmark of 60, giving the target school a deficit of 9 percentage points which is statistically significant at *p* = 0.014 (column 7). The value added by the school has shrunk by 4 percentage points which is 7% of the national baseline (column 14).

Thus, even though these students had the first year of their 2-year course with the 10 a.m. start, reverting to the 8:50 a.m. start for their second year is associated with a reduction in success relative to the national baseline and with a reduction in the value added by the school.

**Figure 2:**
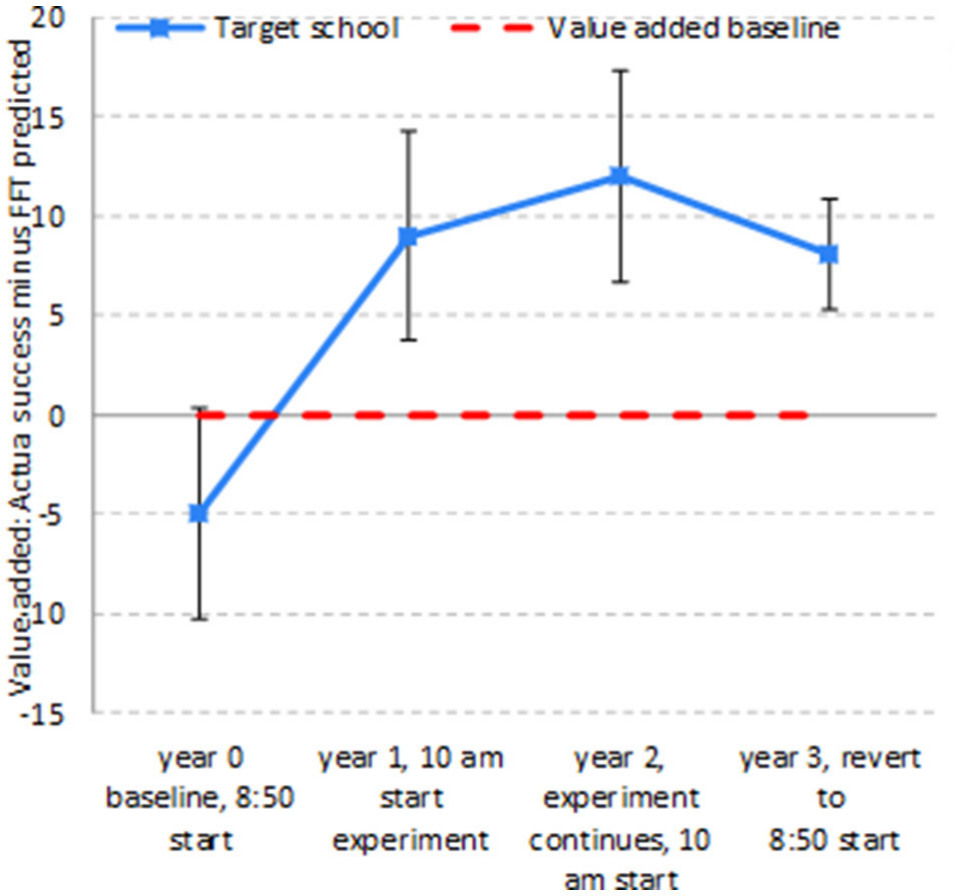
Academic success: value added by target school.

The whole pattern for the combined results of the field experiment and the natural experiment is clear in Figure 2.

In Year 0, student success in the target school was actually 5 percentage points below what would have been expected in light of the students' prior performance (FFT). Then the field experiment began. The introduction of the 10 a.m. start halfway through the Year 1 cohort's course lifted the school's value added to 9 percentage points above the expected success percent. There was a further small rise to a value added of 12 percentage points in Year 2 for the cohort which had experienced the 10 a.m. start for both years of their course. The field experiment then ended. The natural experiment then reverted the start time to 8:50, so students in the Year 3 cohort had the first year of their course with the 10 a.m. start and the second year of their course with the 8:50 start. Value added was still positive for this cohort, but had fallen by 4 percentage points compared to the previous cohort which had 2 years of 10 a.m. starts.

## Discussion

Based on this study, moving school start times later so that they are better aligned with adolescent sleep and chronotype patterns is practical and beneficial. Following a change to a 10:00 a.m. school start time, rates of absence due to illness in students aged 13–16 reduced, and academic performance of students aged 14–16 significantly improved. When the school start time was returned to 8:50 a.m., these benefits were abolished. These findings suggest that the general policy recommendation to start high schools no earlier than 08:30 a.m., while helpful, should not be taken as justification to exclude consideration of much later starting times. As the 8:50 a.m. would already be considered appropriate using the 8:30 a.m. recommendation, this study shows further improvements can be made when starting times are much later, in this case 10:00 a.m.

The absence due to illness rate data are consistent with a direct benefit of later school starting times on student health. The differentiation in recording absence and absence due to illness in England offers a more precise measure of illness and a large national data set. Following initiation of the 10:00 a.m. start, illnesses decreased in the second year by over 50%. With the return of an 8:50 a.m. these benefits were reversed, with illness increasing by of 30%. Academic performance of students aged 14–16 also improved with a 10:00 a.m. start. Having 2 years of later start times was particularly beneficial; showing a 12-percentage point gain in the value added that is statistically significant at *p* < 0.0005 and amounts to 20% of the national benchmark. There is no reason to believe that these outcomes reflected motivation changes. The pupils were studying for their final national exams, which determine their eligibility for continued study and ultimately college or university, or their competitiveness in the jobs market. These were not study-related tests but the real, once-in-lifetime exams that have a major influence on the children's futures and thus would be equally motivated. While there are several possible explanations for the poor performance of students in similar low socioeconomic status areas, the possible impact of sleep restriction linked to early school starts has rarely been considered.

One of the objections raised to changing school start times is that the change is impractical and cannot overcome other barriers, such as bus timetables or sports program scheduling. While the English legal framework makes changing to much later start times a formal process any school can undertake (and therefore more practical than in some other countries), moving all of this school's schedules later produced no practical difficulties. By choosing a 10 a.m. start time, the school aimed to provide benefits for the largest proportion of children possible, given the inter-individual range in phase delays that children experience means some substantially delayed children would still be waking too early in their circadian cycle (Iglowstein et al., 2003; Kelley et al., 2015). Even later start times might address this issue, but 10 a.m. was considered a reasonable compromise between maximizing biological benefits for most children while remaining practical. The national US recommendation that middle and high schools should start after 8:30 a.m. is a clearly justified positive step, although the evidence in this study suggests a much later start of 10 a.m.—even when replacing a school start later than the 8:30 a.m. recommendation—brings additional benefits. A recent study of university students aged 18–19 found that later starting times (after 11 a.m. or 12 noon) were optimal and much later than an 8:30 a.m. start recommended for High School students of 17–18. The study also found that using a fixed time for all students would disadvantage one or more chronotypes, and evening chronotypes in particular if starts were early (Kelley et al., 2015; Evans et al., 2017). Thus, even with the benefits accrued using a 10 a.m. wake time for all students, this approach does not address the wider variation of wake times in different chronotypes.

The limitations in this study include small sample sizes in some measures, a focus mainly on illness, and an inability to measure students' sleep duration. Although other studies have shown sleep improvements following less substantial interventions, some have tracked sleep improvements over 4 years and found that improvements persist (Borlase et al., 2013; Wahlstrom et al., 2014). The English location and measures of academic performance are difficult to contextualize with previous research using U.S. state-wide testing and graduation data. The nationally available data on illness on a school-by-school basis, focus on students in mid-adolescence and the starting time of 10:00 a.m. are, we believe, unique in this research field. A larger, more detailed study is needed of school starts after 9:00 a.m., including those that are later than 10:00 a.m. There is preliminary evidence that such changes can have benefits, particularly for older students (Carrell et al., 2011).

The school-level improvements in performance in this study, if more widely replicated, should be examined from economic and educational perspectives. For example, expenditure to reduce the English attainment gap between rich and poor students reached more than one billion pounds between 2010 and 2015, and yet had relatively little impact compared to the gains made following the 10 a.m. school start time change in this study. The change to smaller schools in key U.S. cities such as New York City have been thoroughly researched after additional expenditure of over a billion dollars to improve performance (Schneider et al., 2007; Stiefel et al., 2015). Other educational policy changes including creating new kinds of school such as Charter, Academy, and Science, Technology, Engineering and Math (STEM) schools, increasing the duration of school times, curriculum and test changes, or No Child Left Behind also have very high expenditure but minimal impact and little scientific rigor in evaluating their effects. In contrast, changing to later start times is a very cost-effective intervention to raise educational standards with substantial scientific backing (Jacob and Rockoff, 2011; Snow, 2015; Hafner et al., 2017).

The key finding in this study is reduced absences due to illness by over 50% compared to national rates (Table 1, columns 12 and 13). This huge change is both practically important and highly significant statistically (*p* < 0.0005 and Cohen's d for the change is 1.07, a large effect size). The broader impact of later starts on specific aspects of adolescent health, such as sleep duration and quality, mental health, and social development were not assessed, although other studies have shown potential impacts (de Souza and Hidalgo, 2014; Meltzer et al., 2014; Minges and Redeker, 2016). Additional research into much later starts should measure both actual sleep patterns and optimal performance times for individual students. The most important area for further research may be the impact of later starts on areas of social behavior development and mental health. For example, the daily sleep loss of two or more hours per day imposed by early school starts (which cannot be recovered with 10 or more extra hours of sleep at weekends), may put those with a genetic predisposition to a mental illness at greater risk given that direct links between sleep of <6 h and gene expression have been established (Möller-Levet et al., 2013). Sleep deprivation is also associated with adolescents being less perceptive readers of human emotions (van der Helm et al., 2010; Guadagni et al., 2014), during a period of greater sensitivity to sociocultural signals (Blakemore and Mills, 2014) and related brain developments in adolescence. These interrelated factors of significant sleep deprivation, genetic predisposition, the high prevalence of the onset of mental illness during adolescence for a range of disorders (Schmitt et al., 2014) and less a perceptive reading of sociocultural signals, may impact on levels of mental illness and emotional disorders in adolescence (Wulff et al., 2010, 2012).

Using a research-based approach to determine a school starting time for 13 to 16-year-old students led to the implementation of a 10 a.m. school starting time. This later starting time had a substantial benefit for rates of illness and academic performance. A research-based approach to school starting times is clearly replicable in different contexts, cultures, and countries. More importantly, a post-9:00 a.m. school start strategy is one with few costs and many potential benefits which start to accrue, quite literally, overnight. Application of sleep research in this way demonstrates the powerful impact on society and individuals of making evidence-based policy changes.

## Ethics statement

The Innovation Trust and School Governing Body approved the change in starting times in compliance with English Educational Law. Parents and students were consulted during the change as legally required.

## Author contributions

PK, SL, and JK: study design; JK and ME: data analysis; PK, SL, JK, and ME: writing and critical review of manuscript.

### Conflict of interest statement

SL has had a number of commercial interests in the last 12 months (2016–17). None are directly related to the research or topic reported in this paper but, in the interests of full disclosure, are outlined below. SL has received consulting fees from the Atlanta Falcons, Atlanta Hawks, BHP Billiton and Slingshot Insights; has current consulting contracts with Akili Interactive; Consumer Sleep Solutions; Delos Living LLC; Environmental Light Sciences LLC; Headwaters Inc.; Hintsa Performance AG; Light Cognitive; Mental Workout; OpTerra Energy Services Inc.; Pegasus Capital Advisors LP; PlanLED; and Wyle Integrated Science and Engineering; has received unrestricted equipment gifts from Biological Illuminations LLC, Bionetics Corporation and F. Lux Software LLC; royalties from Oxford University Press; and has served as a paid expert in legal proceedings related to light, sleep and health. He holds a patent through Harvard University and Brigham and Women's Hospital for “Systems and methods for determining and/or controlling sleep quality.” He is a Program Leader for the CRC for Alertness, Safety and Productivity, Australia. The other authors declare that the research was conducted in the absence of any commercial or financial relationships that could be construed as a potential conflict of interest.

## Footnotes

^1^Fisher Family Trust (2016). Available online at: www.fft.org.uk (Accessed December 2016).
